# Effect of Kidney Shipping and Prolonged Cold Ischaemia Time on Kidney Paired Donation and Living Donor Kidney Transplantation: A Structured Narrative Review

**DOI:** 10.7759/cureus.111041

**Published:** 2026-06-17

**Authors:** Pranjal Kashiv, Swati S Jha, Manish Balwani, Anil Kumar, Khushboo Saxena, Maulik A Patel, Jigar Shrimali, Pratik Das, Kapil V Gothwal, Vivek Kute

**Affiliations:** 1 Nephrology, All India Institute of Medical Sciences, Nagpur, Nagpur, IND; 2 Clinical Research, Case Western Reserve University, Ohio, USA; 3 Nephrology, Saraswati Kidney Care Center, Nagpur, IND; 4 Nephrology, Jawaharlal Nehru Medical College, Wardha, IND; 5 Community Medicine, National Organ and Tissue Transplant Organization (NOTTO), Ministry of Health and Family Welfare, Government of India, New Delhi, IND; 6 Nephrology, Institute of Kidney Diseases and Research Centre, Ahmedabad, IND; 7 Nephrology, Shankus Hospital, Mehsana, IND; 8 Nephrology, Renus Kidney Hospital, Ahmedabad, IND; 9 Nephrology, Rabindranath Tagore International Institute of Cardiac Sciences, Kolkata, IND; 10 General Surgery, Topiwala National Medical College and Bai Yamunabai Laxman Nair Charitable Hospital, Mumbai, IND

**Keywords:** cold ischaemia time, delayed graft function, kidney paired donation, living donor kidney transplantation, organ shipping

## Abstract

Living donor kidney transplantation has traditionally been carried out within a single centre, keeping cold ischaemia time very short. The growth of kidney paired donation, which frequently matches donors and recipients across different centres, has made organ shipping and substantially longer cold storage routine, raising the question of whether living donor kidneys remain resilient under conditions known to injure deceased donor organs. This structured narrative review brings together the contemporary evidence on shipping and prolonged cold ischaemia in adult living donor transplantation, with particular attention to paired exchange, a context not previously examined in a dedicated synthesis. The evidence points to a consistent but modest relationship between longer cold ischaemia and delayed graft function, while the absolute risk stays low and longer-term graft and patient outcomes are largely preserved, albeit on the basis of observational data alone. These findings support current paired donation and shipping practices, including long-distance transport. Cold ischaemia time should nonetheless be minimised wherever practical, particularly for older donors and highly sensitised recipients, through matching algorithms that account for cold ischaemia time, selective donor travel, and machine perfusion when prolonged storage is anticipated.

## Introduction and background

Kidney transplantation remains the optimal renal replacement therapy for people with end-stage renal disease, offering clear advantages in survival, quality of life and cost over maintenance dialysis. Among transplant modalities, living donor kidney transplantation consistently delivers superior short- and long-term outcomes compared with deceased donor transplantation [1]. This advantage has several interlocking sources: the carefully selected health of the living donor, the elective and meticulously planned nature of the procedure, the absence of the haemodynamic and inflammatory insults that accompany brain death or circulatory arrest in deceased donors, and the brief cold ischaemia time achievable when donor nephrectomy and recipient implantation occur within the same institution and the same operative session [1,2].

Short cold ischaemia time has long been regarded as an almost defining feature of living donor practice. A large deceased donor literature study has established that prolonged cold storage drives mitochondrial injury, oxidative stress, endothelial dysfunction and a cascade of inflammation that culminates in tubular injury, delayed graft function and, at its most severe, primary non-function [3]. Living donor kidneys, procured under controlled conditions and implanted within the same surgical session, have historically been spared this injury, with cold ischaemia times of under one hour the norm in conventional same-centre practice [2].

The architecture of living donor transplantation has, however, changed considerably over the past two decades. The recognition that many willing living donors are immunologically incompatible with their intended recipients, whether through ABO blood group mismatch or donor-specific anti-human leucocyte antigen antibodies, gave rise to kidney paired donation as an organised clinical strategy. In its simplest form, paired donation exchanges organs between two incompatible donor-recipient pairs. As computer matching algorithms matured and regional and national registries were established, paired donation expanded to encompass multi-way exchanges, non-directed altruistic donor-initiated chains and, more recently, voucher-based arrangements that decouple the timing of donor and recipient surgery [1,4].

This expansion carries a logistical consequence: when the donor and recipient are matched across institutional and geographic boundaries, either the organ or the donor must travel. In the United States, the United Kingdom and Australia, the donor kidney is shipped to the recipient centre while the donor remains at home [5-7]. In the National Kidney Registry experience reported by Treat and colleagues, shipped paired donation transplants had a median cold ischaemia time of 9.3 hours, ranging from roughly 15 minutes to almost 24 hours, against a median of under one hour in non-shipped, non-paired living donor transplants at the same centres [5].

This evolution raises a question that is at once clinical and biological. If cold storage is harmful in the deceased donor setting, where the relationship between ischaemia time and graft outcome is well established, can living donor kidneys be presumed to tolerate the durations of cold ischaemia that paired exchange logistics now routinely impose? Or does the inherent quality of the living donor organ, set against the favourable physiology of an elective recipient, confer enough resilience to render cold ischaemia time of marginal clinical importance? The answer bears directly on matching algorithm design, the willingness of programmes to engage in long-distance exchange, the counselling of donors and recipients about organ transport, and the potential role of hypothermic and normothermic machine perfusion in the living donor setting [1,3,6].

A systematic review and meta-analysis by van de Laar and colleagues in 2022 addressed the broader question of cold ischaemia time and living donor outcomes, identifying eight eligible studies and reporting a pooled odds ratio for delayed graft function of 0.61, favouring cold ischaemia time under four hours [1]. That review did not, however, stratify by paired exchange exposure and was constrained by the categorical heterogeneity of its included studies. No focused, contemporary review has yet addressed this question specifically within the paired exchange setting, and the present review advances on the earlier syntheses in three respects. First, it takes shipping and the paired exchange context as its explicit focus, rather than cold ischaemia time as an undifferentiated variable across all living donor transplantation. Second, it incorporates the major analyses that have appeared since that review.

Third, because the included studies define cold ischaemia time and shipping too unevenly for a single pooled estimate to be meaningful, it synthesises the evidence narratively under the Synthesis Without Meta-analysis (SWiM) reporting guideline [8], with the full rationale for not pooling set out in the Methods. The aim is to identify, appraise and synthesise the current evidence on how kidney shipping and prolonged cold ischaemia time affect outcomes in adult living donor kidney transplantation, with particular attention to transplants performed through kidney paired donation.

## Review

Methodological approach

Relevant studies were identified by searching PubMed/MEDLINE and Embase to December 2025 using the terms 'cold ischaemia time', 'kidney paired donation', 'living donor kidney transplantation', 'organ shipping' and 'delayed graft function', with titles, abstracts and full texts screened against the criteria below and data extracted into the summary tables. This is a structured narrative review prepared in accordance with the SWiM reporting guideline [8], with the certainty of evidence for each outcome appraised and reported narratively. Studies were eligible if they examined adult recipients (aged 18 years or older) of living donor kidney transplants, with or without kidney paired donation, and reported at least one outcome of interest in relation to kidney shipping, cold ischaemia time (analysed categorically or as a continuous variable) or participation in a paired exchange likely to lengthen cold storage. The primary outcome was delayed graft function; secondary outcomes were acute rejection, all-cause graft loss, death-censored graft failure, patient survival, estimated glomerular filtration rate, serum creatinine and primary non-function. Randomised trials, prospective and retrospective cohorts, registry analyses and paired-kidney analyses were eligible; case reports, series of fewer than 50 recipients, reviews, editorials, abstracts without full publication, modelling and animal studies, and purely logistical reports were excluded. Only English-language publications were considered. Where more than one analysis drew on the same registry, the most recent and most rigorous was treated as the primary source for each outcome and the related analyses were considered alongside it rather than counted again. The broader meta-analyses and the same-donor paired-kidney analysis are used as supporting evidence rather than counted among the primary studies.

A quantitative meta-analysis was deliberately not undertaken. The studies define cold ischaemia time in incompatible ways - some contrasting under four hours with more than four, some using narrow categories such as 0-2, 2-4, 4-8 and 8-16 hours, others modelling it as a continuous per-hour variable - so that a single pooled estimate would obscure rather than clarify. They also address related but distinct questions, some treating cold ischaemia time as the principal exposure and others treating shipping as the exposure, with cold ischaemia time merely one covariate. Finally, several analyses share underlying registries, notably the National Kidney Registry, the Scientific Registry of Transplant Recipients and the Australia and New Zealand Dialysis and Transplant Registry, so pooling them would risk counting the same recipients more than once. Effect estimates from individual studies are therefore presented in summary tables organised by outcome and exposure, and the synthesis describes the direction and consistency of effect and examines modification by donor, recipient and operational factors.

Characteristics of included studies

Twelve studies (Table 1), published between 2007 and 2024, span the entire modern era of organised kidney paired donation. They are predominantly retrospective registry analyses, with one prospective multicentre cohort and no randomised controlled trials, and draw chiefly on the Scientific Registry of Transplant Recipients and the National Kidney Registry in the United States, the Australia and New Zealand Dialysis and Transplant Registry, and the United Kingdom Living Kidney Sharing Scheme. Cohorts range from 56 recipients in the early feasibility study of Segev and colleagues [9] to 48,498 in the registry analysis of Gill and colleagues [10], with median follow-up from roughly one year to ten years.

**Table 1:** Characteristics of included studies on kidney shipping and cold ischaemia time in living donor kidney transplantation. AKX: Australian Kidney Exchange; ANZDATA: Australia and New Zealand Dialysis and Transplant Registry; CIT: cold ischaemia time; DDKT: deceased donor kidney transplantation; DGF: delayed graft function; KPD: kidney paired donation; LDKT: living donor kidney transplantation; NKR: National Kidney Registry; SRTR: Scientific Registry of Transplant Recipients; UKLKSS: United Kingdom Living Kidney Sharing Scheme; UNOS: United Network for Organ Sharing.

Study (first author, year)	Country/Source	Design	Population (n)	Exposure definition	Follow-up
Simpkins, 2007 [2]	USA/UNOS	Retrospective registry	38,467 LDKT (1990–2005)	CIT 0–4 h vs 4–8 h	≥one year
Segev, 2011 [9]	USA, multicentre	Prospective, multicentre	56 KPD transplants	Feasibility of organ transport	Short-term
Allen, 2016 [7]	Australia/AKX	Retrospective KPD cohort	100 KPD transplants (initial AKX experience)	Shipping distance and CIT	Short to medium term
Krishnan, 2016 [11]	Australia/NZ/ANZDATA	Retrospective registry	3,717 LDKT (1997–2012); 224 with CIT >4–8 h	CIT 1–4 h vs >4–8 h; AKX subgroup	Median 6.6 years
Nath, 2016 [12]	UK	Retrospective registry	9,156 LDKT	CIT 0–4 h vs >4–8 h	One, three, five years
Gill, 2017 [10]	USA/SRTR	Retrospective registry	48,498 LDKT (2005–2015)	CIT 0–2 vs 2–4 vs 4–8 vs 8–16 h; KPD subgroup	Up to 10 years
Treat, 2018 [5]	USA/SRTR+NKR	Retrospective registry, linked	1,267 shipped KPD vs 205 in-centre KPD vs 4,800 non-KPD	Shipped vs non-shipped KPD; CIT continuous	Multi-year
Mogulla, 2019 [13]	Australia/NZ/ANZDATA	Retrospective registry	LDKT 2004–2015 (paediatric excluded)	Risk factors for DGF in LDKT including CIT	Short to medium term
Nassiri, 2020 [14]	USA/NKR	Retrospective KPD registry	2,363 shipped LDKT (Feb 2008–May 2018)	CIT ≥16 h vs <16 h (“oldest and coldest”)	Up to seven years
van de Laar, 2021 [6]	UK/UKLKSS	Retrospective registry	9,967 LDKT under UKLKSS	CIT >10 h vs shorter; UKLKSS shipping context	Multi-year
Chipman/Roll, 2022 [15]	USA/SRTR+NKR	Retrospective registry, linked	154 originally compatible pairs in KPD	Compatible-pair KPD (median CIT 10 h) vs traditional KPD	Multi-year
Foley, 2023 [16]	Canada	Retrospective cohort	LDKT and DDKT combined	Combined warm and cold ischaemia time	Multi-year

Effect of cold ischaemia time and shipping on transplant outcomes

Delayed Graft Function (Primary Outcome)

Eleven of the twelve studies reported delayed graft function, and they converged on a single qualitative pattern: a graded rise in delayed graft function with increasing cold ischaemia time, set against an absolute incidence that stayed low across every stratum encountered in contemporary paired exchange (Table 2). The earliest substantial analysis, by Simpkins and colleagues in 38,467 UNOS living donor transplants, showed the adjusted probability of delayed graft function rising from 4.7% at a cold ischaemia time of 0-2 hours to 8.3% at 4-6 hours, and offered a cautious endorsement of organ transport for paired exchange [2]. Four pivotal analyses published between 2016 and 2018 then shaped the contemporary view. Krishnan and colleagues, in 3,717 Australian and New Zealand transplants with a median follow-up of 6.6 years, found a significant increase in delayed graft function beyond four hours of cold ischaemia and identified donor age as an effect modifier [11]. Nath and colleagues, in more than 9,000 United Kingdom transplants, reported a mean absolute difference of 3.1% (95% CI 0.8-5.5) in delayed graft function between the 4-8-hour and the under-two-hour categories [12].

**Table 2:** Effect estimates for delayed graft function across included studies. aOR: adjusted odds ratio; CIT: cold ischaemia time; DGF: delayed graft function; KPD: kidney paired donation; LDKT: living donor kidney transplantation; UKLKSS: United Kingdom Living Kidney Sharing Scheme; UNOS: United Network for Organ Sharing.

Study	Exposure (CIT or shipping)	DGF incidence (%)	Adjusted effect estimate (95% CI)
Simpkins, 2007 [2]	CIT 4–8 h vs 0–4 h	4.7% (0–2 h) vs 8.3% (4–6 h)	Increased odds of DGF with longer CIT; cautious endorsement of organ transport feasibility
Krishnan, 2016 [11]	CIT >4–8 h vs 1–4 h	Higher DGF in the longer-CIT group; donor age effect modifier	Significantly higher DGF with prolonged CIT; the AKX subgroup showed no detrimental effect of shipping within the operational range
Nath, 2016 [12]	CIT 4–8 h vs <2 h	Mean difference 3.1% (95% CI 0.8–5.5)	Graded increase in DGF; significant rise in 12-month creatinine in the longest category
Gill, 2017 [10]	CIT 0–2 vs 2–4 vs 4–8 vs 8–16 h	3.3% vs 3.9% vs 4.3% vs 5.5% (monotonic)	8–16 h vs 0–2 h: aOR 1.47 (95% CI 1.05–2.05); other categories not significant
Treat, 2018 [5]	Shipped KPD vs non-shipped (per hour CIT)	5.1% shipped vs lower in non-shipped	Per additional hour CIT: aOR 1.05 (95% CI 1.02–1.09; p<0.01)
Nassiri, 2020 [14]	CIT ≥16 h vs <16 h (shipped KPD)	5.2% overall; 6.0% had CIT ≥16 h; no significant difference between strata	No significant association between CIT ≥16 h and DGF across supplementary strata
van de Laar, 2021 [6]	CIT >10 h vs shorter (UKLKSS)	Higher DGF in longest CIT category	Increased DGF with prolonged CIT; graft survival excellent even with CIT >10 h
Chipman/Roll, 2022 [15]	Compatible-pair KPD (median CIT 10 h) vs traditional KPD	1% DGF in compatible-pair recipients	No significant difference in graft failure or mortality vs traditional KPD
Mogulla, 2019 [13]	Risk factors for DGF in LDKT	DGF a predictor of poor long-term graft function	Prolonged CIT among identified risk factors for DGF
Segev, 2011 [9]	Transported (shipped) LDKT; median CIT 7.2 h	0% (0/56)	Not estimated (descriptive); 0% vs 4% UNOS LDKT benchmark
Allen, 2016 [7]	Shipped (CIT 6.8 h) vs non-shipped (CIT 2.6 h) KPD	2% (2/100)	Not estimated (descriptive); no difference between shipped and non-shipped recipients

The most granular categorical analysis came from Gill and colleagues in 48,498 SRTR living donor transplants, who described a monotonic rise in incidence across four strata, 3.3% at 0-2 hours, 3.9% at 2-4, 4.3% at 4-8 and 5.5% at 8-16 hours, yet found only the longest category significantly associated with delayed graft function in multivariable analysis (adjusted odds ratio 1.47, 95% CI 1.05-2.05), with no detectable increase in all-cause or death-censored graft loss over follow-up to 10 years [10]. The dedicated examination of shipping itself was provided by Treat and colleagues using linked SRTR and National Kidney Registry data; across 1,267 shipped paired exchange transplants (median cold ischaemia time: 9.3 hours, range: 15 minutes to 23.9 hours), each additional hour raised the adjusted odds of delayed graft function by 5% (adjusted odds ratio 1.05, 95% CI 1.02-1.09, p<0.01), while showing no association with all-cause graft failure (adjusted hazard ratio 1.01, 95% CI 0.98-1.04) [5]. Nassiri and colleagues pushed the operational range to its limit, reporting that in 2,363 shipped transplants, 6% with cold ischaemia of 16 hours or more, even the combination of advanced donor age and very prolonged cold storage produced no significant excess of delayed graft function or death-censored graft failure, a finding that extended the apparent boundary of tolerability well beyond the conventional eight-hour mark [14]. The United Kingdom Living Kidney Sharing Scheme cohort of 9,967 transplants likewise showed more delayed graft function at the longest cold ischaemia times but excellent overall graft survival and led van de Laar and colleagues to argue for building cold ischaemia time into the matching algorithm itself [6].

Two further analyses sharpen the picture. Chipman, Roll and colleagues studied 154 originally compatible pairs who entered paired exchange voluntarily to obtain a younger donor or a better human leucocyte antigen match; the actual donor was indeed younger (median 39 versus 50 years), recipients accepted a median cold ischaemia time of around 10 hours, delayed graft function occurred in just 1%, and graft failure and mortality were no different from traditional paired exchange [15]. A same-donor paired-kidney analysis by Husain and colleagues offers perhaps the cleanest demonstration of dose-response: across 25,831 pairs of kidneys from the same donor experiencing different cold ischaemia times, the longer-stored kidney carried an adjusted relative risk of delayed graft function of 1.17 (95% CI 1.14-1.21), rising with the within-pair difference from 1.05 (95% CI 1.01-1.10) below five hours to 1.47 (95% CI 1.30-1.66) at 15 to 20 hours [17]. Because the paired design holds donor characteristics constant, it isolates the effect of cold storage itself. Taken together, the direction of effect is consistent across every study, the per-hour magnitude is small (about a 5% increase in adjusted odds), and the absolute incidence of delayed graft function stays low even at the extremes of cold ischaemia examined. Confidence in this conclusion is moderate, limited chiefly by residual confounding inherent to observational data.

Acute Rejection

Three studies reported acute rejection (Simpkins [2], Krishnan [11], Nath [12]), and in aggregate, none showed a statistically significant or clinically meaningful association with prolonged cold ischaemia time. Pooling these three, van de Laar and colleagues reported an odds ratio of 1.17 (95% CI 0.86-1.58, p=0.31) for cold ischaemia under versus over four hours, reinforcing the absence of a rejection signal [1]. Confidence here is limited, constrained by imprecision and by inconsistent definitions of rejection.

Graft and Patient Survival

Eight studies reported graft survival or death-censored graft failure, and the operationally important finding is that the measurable effect on delayed graft function does not consistently carry through to longer-term graft loss within the cold ischaemia ranges of contemporary paired exchange. Gill found no increase in all-cause or death-censored graft loss within sixteen hours over follow-up to 10 years [10]; Treat reported an adjusted hazard ratio of 1.01 (95% CI 0.98-1.04) per hour for all-cause graft failure [5]; and Nassiri found no association even at sixteen hours or more [14]. The pooled estimates of van de Laar favoured shorter cold ischaemia at one and five years (odds ratios 0.72, 95% CI 0.60-0.87, and 0.88, 95% CI 0.79-0.99) but with small absolute effect, and the 10-year difference was not significant (odds ratio 0.84, 95% CI 0.64-1.09) [1]; confidence for the graft-survival conclusion is moderate. Patient survival, reported in two studies, showed no significant association (pooled odds ratios 0.74, 95% CI 0.51-1.07 at one year, and 0.54, 95% CI 0.20-1.45 at five years), with limited certainty owing to imprecision [1].

Graft Function

Three studies reported 12-month function. Nath found adjusted mean serum creatinine values of 122, 124 and 126 µmol/L across cold ischaemia times of under two, two to four and four to eight hours, the difference between extremes reaching significance but of small clinical magnitude [12]; Krishnan found a significantly lower one-year glomerular filtration rate beyond four hours [11], and Simpkins found no significant difference in one-year creatinine [2]. The aggregate signal is a small effect on the 12-month function whose long-term clinical significance remains uncertain (confidence limited). The overall certainty of evidence across outcomes is summarised in Table 3.

**Table 3:** Summary of findings and certainty of evidence. aHR: adjusted hazard ratio; aOR: adjusted odds ratio; CI: confidence interval; CIT: cold ischaemia time; DGF: delayed graft function; eGFR: estimated glomerular filtration rate; OR: odds ratio.

Outcome	No. of studies	Direction of effect	Principal effect estimate	Certainty of evidence
Delayed graft function	11	Higher DGF with longer CIT; small absolute increment	Per-hour aOR 1.05 (95% CI 1.02–1.09) [5]; pooled OR 0.61 (95% CI 0.49–0.77) favouring <4 h [1]	Moderate
Acute rejection	3	No clinically significant association	Pooled OR 1.17 (95% CI 0.86–1.58, p=0.31) [1]	Low
Graft survival, one year	2	Favours shorter CIT; modest absolute effect	Pooled OR 0.72 (95% CI 0.60–0.87) [1]	Moderate
Graft survival, five years	4	Favours shorter CIT; small absolute effect	Pooled OR 0.88 (95% CI 0.79–0.99) [1]	Moderate
Death-censored graft failure	4	No significant association within CIT ≤16 h	aHR 1.01 (95% CI 0.98–1.04) per hour [5]; no association for CIT ≥16 h [14]	Moderate
Patient survival	2	No significant association	Pooled OR 0.74 (95% CI 0.51–1.07) at 1 y; 0.54 (95% CI 0.20–1.45) at 5 y [1]	Low
Graft function (12-month creatinine/eGFR)	3	Small creatinine rise with longer CIT; significance uncertain	Mean adjusted creatinine 122 vs 126 µmol/L (<2 h vs 4–8 h) [12]	Low

Interpretation and comparison with prior evidence

Across the included studies, encompassing in excess of 160,000 living donor kidney transplant recipients - though with substantial overlap between registry-derived cohorts - three conclusions emerge. First, the absolute risk of delayed graft function in living donor transplantation stays low even at the upper extremes of cold ischaemia time seen in paired exchange, rising from roughly 3% with minimal cold storage to 5-6% at eight to sixteen hours [10]: figures far below the 20-30% routinely reported in deceased donor transplantation [3], and a reflection of the inherent quality of the living donor organ and the elective physiology of its recipient. Second, a small but consistent and biologically plausible per-hour association nonetheless persists, anchored by the Treat estimate (adjusted odds ratio 1.05) [5] and independently echoed by Yao and colleagues across all transplant types (pooled odds ratio for prolonged cold ischaemia time 1.05, 95% CI 1.03-1.07) [18] and by the categorical estimate of van de Laar [1]; the agreement of per-hour and categorical approaches across heterogeneous populations is what lends the finding its robustness. Third, that measurable effect on delayed graft function does not translate into meaningful long-term harm within the operational range of paired exchange, with graft and patient survival broadly preserved on follow-up to 10 years [5,6,10,14].

These conclusions sit comfortably with the 2022 review of van de Laar and colleagues [1]. The present review differs in addressing the paired exchange context explicitly, in incorporating subsequently published analyses, Chipman and Roll [15], Foley [16], and the meta-analyses of Tirtayasa [19] and Yao [18], and in adopting the SWiM framework in recognition of the methodological heterogeneity of the evidence. The 2024 meta-analysis of Tirtayasa and colleagues, pooling 13 studies and 113,261 patients, placed prolonged cold ischaemia time among the significant risk factors for delayed graft function in living donor transplantation [19], while the convergence of the Yao and Treat estimates strengthens confidence in the effect size.

Clinical implications and special populations

The aggregate evidence describes an average effect; the decision to ship a kidney in any individual case turns on donor and recipient characteristics that may modify it. Donor age is the clearest example: Krishnan identified it as an effect modifier [11], consistent with the reduced functional reserve of older donor kidneys, yet Nassiri showed that advanced donor age combined with prolonged cold ischaemia within the National Kidney Registry range produced no significant excess of delayed graft function or graft failure [14]. Older living donors should therefore not be excluded from shipped exchange on age alone, though individualised assessment remains warranted. For highly sensitised recipients and those with donor-specific antibodies, for whom paired exchange is often the only realistic route to transplantation, Chipman and Roll demonstrated that the longer cold ischaemia of exchange did not worsen graft or patient survival [15], so the immunological benefit comfortably outweighs the modest incremental risk. For ABO- or human leucocyte antigen-incompatible transplants, Tirtayasa identified ABO incompatibility as an independent risk factor for delayed graft function [19]; the interaction between incompatibility, desensitisation and prolonged cold ischaemia has not been studied directly and remains an important gap.

Operational strategies and the South Asian context

Because cold ischaemia time exerts a real, if modest, effect, there is a rational case for minimising cold storage wherever feasible. The most direct lever is to build cold ischaemia time into the matching algorithm, as van de Laar and colleagues advocated after showing that cold ischaemia time varied widely across matched pairs in the United Kingdom programme and could be reduced on average without sacrificing matching efficiency [6]. An alternative is donor travel, used in the Netherlands and Canada, in which the donor is brought to the recipient centre; this preserves the short cold ischaemia profile of same-centre transplantation but shifts the burden onto donors and families, who must travel and be operated upon by an unfamiliar surgical team [1]. Machine perfusion offers a third avenue: in the deceased donor setting, the randomised trial of Moers and colleagues, comparing hypothermic machine perfusion with static cold storage in 336 kidney pairs, found delayed graft function in 70 of 336 perfused kidneys versus 89 of 336 cold-stored (adjusted odds ratio 0.57, p=0.01), with one-year graft survival of 94% versus 90% [3]. Despite this, the role of machine perfusion in living donor paired exchange has scarcely been examined, and its application to shipped kidneys with anticipated cold ischaemia beyond eight hours is a clinically rational, operationally feasible intervention that deserves dedicated study.

The Indian experience has developed along distinctive lines. The first successful three-way kidney paired donation in India was performed at the Institute of Kidney Diseases and Research Centre in Ahmedabad and reported by Kute and colleagues in 2014, achieved through coordinated, simultaneous operations within a single institution that kept cold ischaemia times short [20]. The programme has since grown to encompass multi-centre exchanges, three- and four-way swaps and an emerging national framework [4]. Indian logistical realities, the geographic concentration of high-volume centres, variable inter-city air transport, the absence of a comprehensive national deceased donor allocation framework across all states, and the financial and social burden on donor families, have produced a programme in which donor travel and kidney shipping coexist according to the circumstances of each exchange [4]. The South Asian experience underscores the value of paired donation as a means of expanding access where the alternatives are commercial transplantation, prolonged dialysis or no transplantation at all; in that setting, the modest incremental risk of prolonged cold ischaemia is more than offset by the gains in access [4].

Strengths and limitations

The strengths of this review lie in its pre-specified eligibility criteria, a structured, transparent appraisal of the certainty of evidence for each outcome, and its explicit adoption of the SWiM framework in recognition of methodological heterogeneity; it draws on a substantial evidence base of more than 160,000 living donor recipients across several national registries, with the geographic and operational diversity that lends weight to its conclusions. Several limitations temper the conclusions. As a narrative review, the synthesis reflects the established literature on this question rather than the output of an exhaustive multi-database search, so smaller or less prominent eligible studies may have been missed. All included studies were observational, with no randomised trials of shipping or prolonged cold ischaemia identified, leaving the certainty of evidence constrained by residual confounding. Several analyses drew on overlapping registries, raising the possibility of non-independence; this was managed by retaining the most recent and rigorous analysis as primary for each outcome, though that judgement is inevitably discretionary. The review was restricted to English-language publications, and quantitative pooling was not performed for the reasons set out above; complementary quantitative perspectives are available from the studies by van de Laar [1], Yao [18] and Tirtayasa [19].

## Conclusions

Across more than two decades of registry and cohort evidence, a consistent picture emerges. Prolonged cold ischaemia time carries a real but small association with delayed graft function in living donor transplantation, yet the absolute risk stays low even at the durations that paired exchange now imposes, and longer term graft and patient survival are largely unaffected. These findings support the continued expansion of kidney paired donation, including the long distance shipping of donor organs. Cold ischaemia time should nonetheless be kept as short as is practical, with particular care for older donors and highly sensitised recipients, by building cold ischaemia time into matching algorithms, using donor travel in selected cases, and applying machine perfusion when prolonged storage is anticipated. Dedicated study of machine perfusion in shipped paired exchange, and of how cold ischaemia interacts with immunological risk, remains the clearest priority for future work.
